# An Atypical Ross River Virus Infection in an Australian Army Service Member

**Published:** 2026-02-26

**Authors:** Melissa Graham, Brian Vesely, Cielo Pasay, Wenjun Liu

**Affiliations:** Australian Defense Force Malaria and Infectious Disease Institute, Gallipoli Barracks, Enoggera, Queensland, Australia: CAPT Graham, Dr. Liu, Dr. Pasay; QIMR-Berghofer Medical Research Institute, Brisbane, Queensland: CAPT Graham, Dr. Pasay; Walter Reed Army Institute of Research Engineering and Scientist Exchange Program, Enoggera: MAJ Vesely

Arboviral diseases, transmitted by arthropods such as mosquitoes, represent a significant and ongoing threat to the health, readiness, and mission capability of U.S. military personnel deployed in endemic regions. ^1 , 2^ Ross River virus (RRV), an alphavirus transmitted by mosquitoes, is endemic to Australia and causes an average of 5,000 cases annually. ^3^ RRV is also endemic as well as epidemic in many South Pacific Islands including Papua New Guinea, Solomon Islands, Fiji, American Samoa, New Caledonia, and Cook Islands. ^4^ These countries are frequent locations for U.S. military training and joint operations **(Figure 1)** .

**FIGURE 1.**
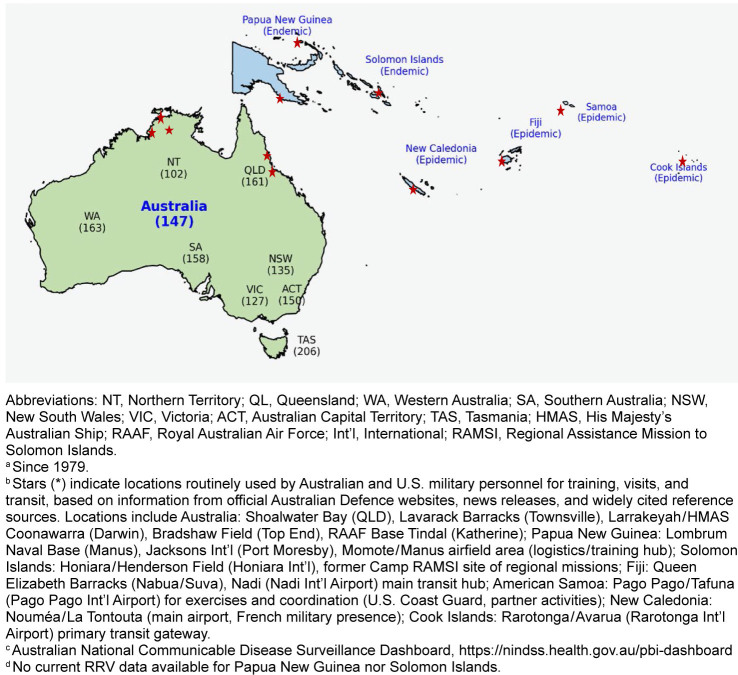
Endemic and Epidemic Countries of Ross River Virus ^a^ and Major Locations of Routine Personnel Training and Visits ^b^ by U.S. Australian and U.S. Armed Forces, with Rates per 100,000 for Australian States and Territories, 2024 ^c,d^

RRV is not a new threat to U.S. military operations. In 1997, an outbreak of RRV-related epidemic polyarthritis (EPA) occurred among 19 U.S. Navy personnel during a joint exercise at the Shoalwater Bay Training Area in Queensland. ^5^ A pre- and post-deployment serum survey of 2,500 U.S. marines deployed to Australia on 6-month training rotations confirmed RRV sero-conversion, indicating RRV local transmission during training deployments. ^6^

U.S. military presence in the South Pacific has increased recently, with several multi-national, joint exercises in response to strategic pressures arising from the expansion of China's southwestern Pacific military presence. More than 35,000 military personnel, including Australian and U.S. forces, and representatives from over 19 nations took part in Exercise Talisman Sabre 2025, the largest military exercise ever held in Australia and the first in Papua New Guinea. ^7^

RRV is the most frequently reported arboviral disease in Australia, with approximately over 63,000 cases recorded in Queensland alone from 1993 to 2020. ^8 , 9^ The ecology of RRV is complex: Over 40 mosquito species have been identified as potential vectors, and more than 18 wild and domestic animal species are suspected as amplifying hosts or reservoirs. ^10^ These factors contribute to unpredictable and seasonal RRV outbreaks. RRV is particularly prevalent in the Northern Territory and Queensland, where human cases are reported year-round. ^11^

Although some RRV infections are asymptomatic or sub-clinical (approximate symptomatic-to-asymptomatic ratio 1:3), symptomatic cases can develop into EPA, a debilitating condition characterized by joint inflammation. Additional symptoms, such as rash, low-grade fever, malaise, myalgia, lymphadenopathy, headache, depression, and fatigue, may accompany EPA. ^12 - 14^ Atypical presentations have been reported, including cases with prolonged or relapsing symptoms, absence of rash or arthritis, neurological involvement, or unusual laboratory findings.

While most symptomatic RRV patients recover within 4–6 weeks, some experience persistent joint or muscle pain and fatigue for months to several years. In a 1996 study of long-term symptomatic cases, at 15 months 51% of respondents still had joint pain, and 45% had persistent tiredness and lethargy ^15^ ; these symptoms were still common up to 30 months after infection. Joint pain is the most common and persistent symptom, with the 4 most common joints affected being ankles (75%), wrist (72%), knees (66%) and fingers (66%). While other affected joints had much lower incidences (4-47%). ^16^ Such cases can pose diagnostic challenges, particularly in military or deployment settings where other vectorborne or febrile illnesses are also possible.

The pathogenesis of persistent arthritis remains unclear, although persistent infection of synovial macrophages has been documented for other alphaviruses. ^17^ RRV-induced arthritis is characterized by inflammatory infiltrates comprised largely of mononuclear cells. Characterization of those infiltrates suggests that monocytes/macrophages are a major constituent of the infiltrate, while immune-histological studies of synovial biopsy samples have also identified CD4 ^+^ and CD8 ^+^ T lymphocytes within inflammatory infiltrates. ^18 , 19^

Symptoms similar to EPA may occur after infection with Barmah Forest virus (BFV), chikungunya virus (CHIKV), Epstein-Barr virus, Rubella virus, and Parvovirus B19. BFV co-circulates with RRV in Australia with approximately 1,600 cases annually. ^20^ Currently, there is no specific antiviral treatment or vaccine for RRV. Clinical management primarily targets symptom relief.

In accordance with the Australian Health Department, definitive laboratory diagnosis of RRV infection requires specific laboratory evidence, including virus isolation or detection of viral RNA (ribonucleic acid) by RT-PCR (reverse transcription-polymerase chain reaction) in serum collected within 6 days of illness onset. Alternatively, diagnosis may be based on serological evidence, such as seroconversion or a greater than or equal to 4-fold increase in immunoglobulin G (IgG) titre, provided there is no corresponding change in antibody levels to BFV. Detection of RRV-specific immunoglobulin M (IgM) in the absence of anti-CHIKV IgM or anti-BFV IgM is also considered confirmatory evidence.

Due to serological cross-reactivity among alphaviruses, particularly BFV and CHIKV, serological diagnosis must be carefully interpreted. Alphavirus-specific IgM antibodies usually last 1–3 months, with levels generally falling subsequently. ^21^ Within 2 weeks of an elevated virus-specific IgM response, a virus-specific IgG level usually becomes detectable, with IgG levels persisting for a long period, likely providing lifelong protection. ^22^

We report here an atypical RRV infection in 2024 in an Australian Army service member. The study was approved by the Australian Departments of Defence and Veterans' Affairs Human Research Ethics Committee (protocol DDVA HREC P204-20). This report serves to promote awareness among medical corps and force health protection officers for consideration of deployment-related RRV disease in differential diagnosis of patients with fever, arthralgia, or rash who have recently deployed to, or conducted exercises in Australia.

## Case Presentation

During a routine pre- and post-deployment serological screening program, a concerning seroconversion was identified in an Australian Defence Force (ADF) service member who had recently returned from a 3-week deployment to Papua New Guinea in late April and early May 2024. The pre-deployment serum sample, collected in early February 2024, was negative for anti-RRV IgG/M and neutralizing antibodies (NAb). The post-deployment serum, however, collected in early May 2024, was positive for both anti-RRV IgG / M and NAb, at a dilution of 1 : 320. Negative serology for anti-BFV NAb ruled out cross-reactivity and supported a definitive RRV infection.

Clinical questioning confirmed strict adherence to mosquito bite prevention measures while deployed, including sleeping indoors with screened windows, wearing a permethrin-treated uniform, and consistent use of mosquito repellent. As a result, she sustained few mosquito bites in Papua New Guinea.

Further investigation revealed that the service member resided in a known RRV hotspot in Brisbane, Queensland—an area with ongoing community transmission. The service member recalled significant mosquito exposure in early February (~15 bites per day), 2 months prior to deployment, and developed monoarthritis in the right wrist on February 13th. Imaging studies (x-ray and ultrasound) found no structural injury, and blood examination was negative for rheumatoid factors or other arthritic markers. MRI (magnetic resonance imaging) in March confirmed right wrist joint inflammation-joint effusion/synovitis **(Figure 2a)** .

**FIGURE 2a.**
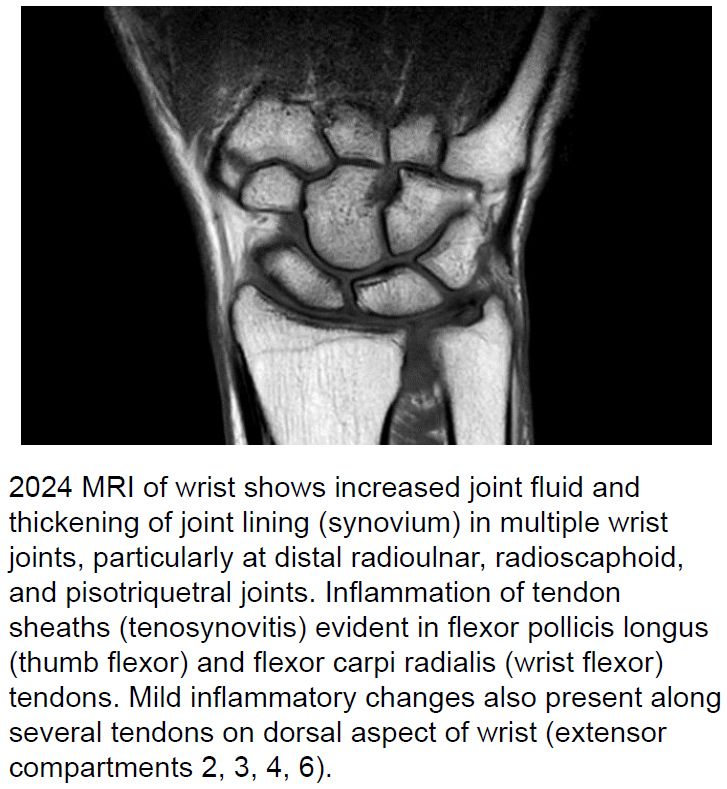
2024 MRI Image of Ross River Virus-Infected Australian Service Member's Right Wrist, Indicating Capsulosynovitis

Despite the service member's back-ground as a laboratory scientist and personal request for RRV testing, her general practitioner dismissed the possibility of RRV infection due to monoarticular involvement.

The service member's symptoms persisted—with manageable pain—until approximately October 2024. Some residual discomfort continued until April 2025, largely triggered by over-use. Follow-up pathology testing for rheumatoid and other arthritic markers was again negative. Additional MRI in April 2025 confirmed mild synovitis **(Figure 2b)** , and corticosteroid injection was administered.

**FIGURE 2b.**
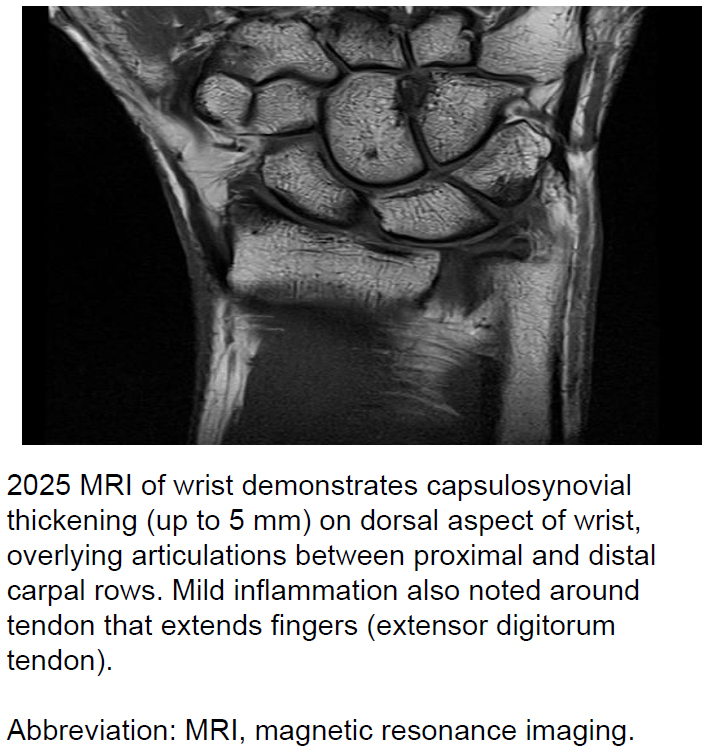
2025 MRI Image of Ross River Virus-Infected Australian Service Member's Right Wrist Indicating Capsulosynovitis

Based on timing, exposure history and serological data, we concluded that the infection likely occurred at the service member's home in Queensland rather than during overseas deployment.

## Discussion

Queensland is the Australian state most affected by RRV, consistently reporting over 1,000 cases annually. A record-breaking number of mosquito samples tested positive for RRV during the 2023-2024 mosquito season (November–April), which coincided with a high number of human RRV cases. Samples from more than 1,225 mosquito traps were tested, with 116 traps yielding positive results, the highest number since 2016, when the current surveillance program began. In the first 4 months of 2024, 2,065 human RRV cases were reported in Queensland, the highest total since the 2019-2020 season. In the second week of March 2024, weekly cases peaked at 333, with over 50% in Southeast Queensland, where incidence was 2.4 times higher than the 5-year average. ^23^

As the Indo-Pacific area becomes a defining theater of 21st century strategic competition, northern Australia, including Queensland and the Northern Territory, has emerged as a crucial area for U.S. force presence and deterrence. ^24^ U.S. military personnel who are deployed to regions where RRV is endemic, including northern Australia, Papua New Guinea, or the Solomon Islands, may be at risk of infection even during short-term exercises or visits. Exposure risk is influenced not only by location but also by timing, duration, and type of activities during deployment.

U.S. military personnel are subject to insect-borne diseases and pest threats that can adversely affect their health and compromise important missions, whether deployed in combat operations, engaged in humanitarian relief, or conducting training. Malaria, as well as flaviviruses such as dengue and West Nile virus, and alphaviruses such as RRV, BFV and CHIKV, along with sandfly fever, scrub typhus, and several tick-borne diseases, continue to pose a significant threat to forces worldwide. The largest outbreak of RRV infection ever recorded, in the Pacific from 1979 to 1980, demonstrates the epidemic potential of the virus. ^25^

The experience of Zika virus outbreaks since 2015 and the explosive CHIKV out-break in China 2025 underscores the serious threat posed to global health by the potential for previously obscure arboviruses to shift from their historical cycles of transmission. ^26 , 27^ This risk is amplified within a mobile population such as the U.S. military.

A further risk is the potential for RRV to be exported to other countries through asymptomatic infected individuals, whether military personnel or civilians. RRV-viraemic travelers have been linked to the spread and epidemics with RRV in the Asia-Pacific region before. ^28^ This risk is of particular concern for the U.S., given the presence of mosquitoes known to be RRV vectors. ^4 , 29^

Australia remains a key partner of the U.S. in joint training operations, with an estimated 2,500 U.S. marines and sailors rotating annually through northern Australia. Additionally, in 2024, approximately 656,000 U.S. citizens traveled to Australia for recreational purposes, highlighting the potential for both military and civilian exposure to these endemic arboviruses. Enhanced surveillance, diagnostic capacity, and medical awareness of RRV, preventive measures during and after deployment must be prioritized in both the U.S. Military Health System and joint force health support planning.

This case underscores the need for heightened clinical awareness among military medical providers. U.S. service members presenting with febrile illness or joint pain after deployment to Australia should be evaluated for RRV as part of a comprehensive differential diagnosis of vector-borne diseases. Because exposure risk may extend beyond deployment sites, both deployment and travel locations should be considered when developing differential diagnoses, which should include arboviruses not endemic to Australia, such as CHIKV, dengue, and Zika virus (ZIKV). A high index of suspicion based on travel location and seasonality is needed to ensure RRV is included in the differential diagnosis.

The U.S. Department of Defence Insect Repellent System is an effective mechanism for protecting military personnel from pests and insect-borne diseases. ^30^ Preventive measures—including the use of DEET (diethyltoluamide)-based repellents, wearing long-sleeved uniforms, and treating uniforms with permethrin—remain critical to force health protection. In addition, medical staff must be aware of the local disease ecology and incorporate arboviral infections into pre-deployment briefings and post-deployment health assessments.
